# Tobacco use by Indian adolescents

**DOI:** 10.1186/1617-9625-1-2-111

**Published:** 2002-06-15

**Authors:** RK Chadda, SN Sengupta

**Affiliations:** 1 Department of Psychiatry, Institute of Human Behavior & Allied Sciences, Delhi, India

## Abstract

Adolescents are the most vulnerable population to initiate tobacco use. It is now well established that most of the adult users of tobacco start tobacco use in childhood or adolescence. There has been a perceptible fall in smoking in the developed countries after realization of harmful effects of tobacco. The tobacco companies are now aggressively targeting their advertising strategies in the developing countries like India. Adolescents often get attracted to tobacco products because of such propaganda. There has been a rapid increase in trade and use of smokeless tobacco products in recent years in the country, which is a matter of serious concern to the health planners. It is important to understand various factors that influence and encourage young teenagers to start smoking or to use other tobacco products. The age at first use of tobacco has been reduced considerably. However, law enforcing agencies have also taken some punitive measures in recent years to curtail the use of tobacco products. This paper focuses on various tobacco products available in India, the extent of their use in adolescents, factors leading to initiation of their use, and the preventive strategies, which could be used to deal with this menace.

## Introduction

Tobacco use in children and adolescents is reaching pandemic levels. The World Bank has reported that nearly 82,000–99,000 children and adolescents all over the world begin smoking every day [1]. About half of them would continue to smoke to adulthood and half of the adult smokers are expected to die prematurely due to smoking related diseases. If current smoking trends continue, tobacco will kill nearly 250 million of today's children [2,3].

India is the second most populous country in the world. It is a secular country but the Hindus form the majority. Hinduism traditionally advocates abstinence from all intoxicants. Even then, India is the third largest producer and consumer of tobacco in the world. The country has a long history of tobacco use. Tobacco is used in a variety of ways in India; its use has unfortunately been well recognized among the adolescents [4-15]. Tobacco addiction of a large number of adults has been initiated during the adolescence [2,4,13,16-18].

Considering the enormous health complications associated with tobacco use, it is of utmost importance to understand the factors leading to its use and to plan strategies to reduce its intake. This is especially relevant for the developing countries like India, where tobacco use continues to be common notwithstanding the recognition of harmful consequences of its usage. This paper reviews the patterns of tobacco use in India, its prevalence in Indian adolescents, role of psychosocial factors in initiation and prevention, and the steps taken by the State to control its use.

## Historical Aspects

Human beings have been using tobacco since 600 A.D. [19]. It was introduced in Europe by Columbus who came to know about it from the Caribbeans during his historical journeys. It was introduced in India by the Portuguese [20]. Earlier, tobacco was generally smoked using different types of pipes or as cigars or was consumed orally (smokeless tobacco). Paper cigars and cigarettes were introduced in the mid nineteenth century. Nicotine was isolated from the tobacco leaves as early as 1828 A.D. [21].

Harmful effects of tobacco have been recognized over the last 1000 years. Historically, three contemporary rulers, King James I of England, Shah Abbas of Persia and the Mughal emperor Jahangir of India (father of Shah Jehan, the builder of Taj Mahal) in 16th century had noticed the harmful effects of tobacco and tried to ban it. King James had commented on smoking as being "a custom loathsome to the eye, hateful to the nose, harmful to the brain, and dangerous to the lungs". Jahangir had even passed orders prohibiting smoking tobacco. Khalil Pasha was more stringent and had passed a prohibitory decree against tobacco smoking that "anybody caught smoking tobacco would have his lips cut and eyes taken out". In 1014 Hijri, Russia had also passed certain regulations against smoking [20,22,23].

India has a distinct tradition of enforcements against tobacco use being initiated by the past kings (like Jahangir) and the religious leaders at different times in the history. The great Indian Sikh Guru Gobind Singh prohibited smoking for the members of the Sikh community. He said, "Wine is bad, Indian hemp (bhang) destroys one generation, but tobacco destroys all generations" [22,23]. Tobacco use has been considered a religious taboo by the Sikhs since then. However, despite historical attempts to legally ban tobacco, its use has continued to grow in popularity as a nonproductive pastime [21].

In the modern medicine, its harmful effects have been recognized over the last 4 decades [19,24]. Although its use has declined in developed nations in the recent years, it continues to be popular in developing countries [3].

## Patterns of Use

Tobacco is used in a wide variety of ways in India including smoking and smokeless use.

### Smoking Practices

Tobacco is smoked in the forms of **beedis **and cigarettes or by using devices like **hooka, hookli, chhutta, dhumti, or chillum **[17,25,26]. Smoking of cigars and pipes are not common in India, as they are in most western countries.

Cigarette smoking is common in urban areas. Both locally manufactured and imported brands of cigarettes are available. However, because of relatively higher cost of cigarettes as compared to other forms of tobacco, cigarette smoking is more common among the upper and middle socioeconomic classes than among the poor population.

**Beedi **is a cheap smoking stick, handmade by rolling a dried, rectangular piece of temburni leaf (Diospyros melanaxylon) with 0.15–0.25 g of sun-dried, flaked tobacco filled into a conical shape and the roll is secured with a thread. The length of a beedi varies from 4.0–7.5 cm. Beedis are commercially available in small packets.

**Hooka **(a hubble bubble Indian pipe) is an indigenous device, made out of wooden and metallic pipes, used for smoking tobacco. The tobacco smoke passes through water kept in a spherical receptacle, in which some aromatic substances may also be added. Hooka smoking is a common method of socializing among the village folk, especially in the Northern and Eastern parts of India, and is a part of the rural cultural life. Its use is more common among the adults and older generation. However, it is not popular among adolescents, because the adults generally discourage younger population from using hooka.

**Hookli **is a short clay pipe-like device, being about 7 cm long, and is used for smoking tobacco in some parts of the country.

**Chhutta **is a coarsely prepared roll of tobacco (cheroot), smoked with the burning end inside the mouth (reverse chhutta smoking). Its use is prevalent in coastal areas of the province of Andhra Pradesh in southeastern India.

**Dhumti **is a cigar-like product made by rolling tobacco leaves inside the leaf of jackfruit tree. Occasionally, dried leaf of a banana plant is used. Males smoke dhumti in conventional manner, whereas females smoke it in a reverse manner, i.e., keeping the burning end inside the mouth. Dhumti smoking is quite popular in the Goa province of the Western India.

**Chillum **is a conical clay-pipe of about 10 cm long. The narrow end is put inside the mouth, often wrapped in a wet cloth that acts as a filter. This is used to smoke tobacco alone or tobacco mixed with *ganja *(marijuana) in northern parts of the country.

### Smokeless tobacco use

Tobacco is used in a number of smokeless forms in India, which include **betel quid **chewing, **mishri, khaini, gutka**, snuff, and as an ingredient of **pan masala**.

**Betel quid **is a combination of betel leaf, areca nut, slaked lime, tobacco, catechu and condiments according to individual preferences. Tobacco is an optional component. Its use is prevalent all over India. However, there are differences in the components used in different regions of India.

**Khaini **consists of roasted tobacco flakes mixed with slaked lime. This mixture is prepared by the user keeping the ingredients on the left palm and rubbing it with the right thumb. The prepared pinch is kept in the lower labial or buccal sulcus. Its use is common in eastern India.

**Mawa **is a mixture of areca nut, tobacco and slaked lime and is chewed. Its use is common in rural areas of Gujarat province. It is quite popular among the young population of ages 15–19.

**Snuff **is a black-brown powder obtained from tobacco through roasting and pulverization. Snuff is used via nasal insufflation and is popular in eastern parts of the country. It is also applied on the gum by finger (this practice is usually initiated as a dentifrice) in the Western India, where it is known as bajar and mishri.

**Gutka **is a manufactured smokeless tobacco product (MSTP), a mixture of areca nut, tobacco and some condiments, marketed in different flavors in colorful pouches.

**Pan masala **is a betel quid mixture, which contains areca nut and some condiments, but may or may not contain tobacco. The mixture is chewed and sucked. Unlike cigarettes, tax levied on pan masala is low. Low cost and not being associated with smoke have led to an enormous increase in the use of all types of areca nut and smokeless tobacco among the Indian population including adolescents. It has also been promoted as a "post meal mouth freshener", making it quite popular. Initially, it was more popular in the Northern India, but with a massive advertising, it is now being used all over the country. Pan masala is as harmful as smoking, although the nature of harmful effects are different. Its use has been associated with high risk of oral cancer and submucus fibrosis in mouth, which also has a high potential for cancer development. It is made by the use of waste tobacco, mid-ribs of tobacco leaves and floor sweepings from cigarette factories. It is available in the forms of small packets and cans, sold at affordable prices with attractive, shiny colored wrappings [5,25,27,28].

Beedis, khaini, and mishri are commonly used in rural areas and cigarettes are mainly used in urban areas. Pan masala was initially popular in the urban segment only, but over the last few years, it has been consumed in rural areas as well.

According to the most recent Government of India's National Sample Survey data, there are 184 million tobacco consumers in India. About 40% of them use smokeless tobacco, 20% consume cigarettes, and another 40% smoke beedis. Smokeless tobacco use includes *pan *masala and chewing of tobacco in different forms. Tobacco is also smoked using indigenous devices like hooka, chhutta or dhumti in different parts of the country [20]. Thus, in contrast to the other parts of the world, tobacco is used in a variety of ways in India, which include smoking and smokeless tobacco use.

## Extent of the Problem

Adolescents and children are the prime targets of the tobacco industry when recruiting new smokers. About 20 million children of ages 10–14 are estimated to be tobacco-addicted according to a survey done by the National Sample Survey Organization of the Indian Government. To this astounding figure, about 5500 new users are added every day, making two million new users every year [4,27]. Adolescents typically become addicted to nicotine while still being teenagers. Usual interval between the first cigarette consumption and daily smoking is 1–2 year(s). More than half of the adolescent smokers try to quit smoking every year with fewer than 20% being able to quit for a month [18]. The majority of adolescent smokers report withdrawal symptoms when they try to quit [1].

During the last three decades, a number of epidemiological surveys has been conducted in different parts of India to study the prevalence of tobacco use by adolescents. The study populations have included school and college students, medical students and street children [30]:

### Tobacco use among school and college students

Table 1 summarizes some important studies done on school and college students. The prevalence of smoking has been found to vary from 6.9 to 22.5% among the male school and college students. Among the girls, the prevalence is considerably low varying from 0–2.3% [6,11]. In fact, tobacco use, especially smoking, is a relatively new habit among the female students, noticed only during the last 10–15 years.

**Table 1:** Studies on tobacco use among school and college students

Author	Sample	Age of onset	Ever use	Current use	Current daily use	Type of tobacco use
Kapoor et al. [6]	School and college students (N = 1386, M-81.5%, F-18.5%)	5–10 in 36.1% 11–15 in 44.0% 16–20 in 19.9%	12% in total sample; 6% in 13–14 age group; 15% in >18 age group; M = 14.2% F = 2.3%	7.1% (smoked in the previous week)	29% (of all smokers)	Smoking
						
Jayant et al. [11]	Urban school students (N = 1633, M-78.4%, F-21.6%)	--	EM^a ^schools – 22.5% in boys PIL^b ^schools – 6.9% in boys MIL^c ^schools – 13.8% in boys Girls – 1.1%	--	--	86% smokers
						
Gavarsana et al. [12]	College students (N = 599, M-64.6%, F-35.4%)	10–12 in 64% 15 or more in 31%	Smoking in 18% boys; Snuff use in 38% boys and 12% girls	Smoking in 8% boys	--	Smoking and snuff tobacco
						
Singhi et al. [15]	School boys (N = 467)	10–14 in 80%	20% in 12–20 age group	-	42% of all smokers	Beedis, cigarettes

a) EM: English language,

b) PIL: Private Indian language,

c) MIL: Municipal Indian language.

The prevalence figures vary according to the criteria used to define tobacco use habits. For example, in one study ever-smokers constituted 14.2% of the study population and current smokers (defined as having smoked in the last week) formed 7.1% of the study sample [6]. More than 40% of children had started the habit between 10–15 years of age. There was no rural urban difference. Being of male gender, having an age above 15, smoking by a close relative (father, mother, sister/brother) or friends were significantly associated with smoking by the adolescent children. Both the smokers and non-smokers were well aware of the adverse health effects of smoking indicating that mere provision of information on hazards of smoking may not be enough to reduce the prevalence of smoking. Jayant et al. reported a higher prevalence of tobacco abuse in male students from English medium schools*(22.5%) compared to the students in private Indian language schools (6.9%) or municipal Indian language schools (13.8%) [11].

The girls had a very low (1.1%) prevalence of tobacco use. Most boys who were using tobacco were smokers (86%). Significantly higher proportion of boys smoked if their father or best friend smoked. Generally, boys were more sensitive to the best friend's or elder brother's disapproval rather than the parental. They were well informed about the general harmfulness of smoking but there was no knowledge on any specific health hazard.

Although smoking is rare among female students, the use of snuff is not uncommon. Gavarsana et al. found tobacco-snuff-use in 38% of males and 12% of females in a sample population of 599 college students [12].

### Tobacco use among medical students

Most students enter medical schools in India in their late adolescence. A number of researchers in India have investigated the use of drugs and smoking among medical students [5,8,31-33]. Table 2 summarizes these findings. Cigarette smoking has been found to be as common among medical students (17.6–33.2%) as among other college students. Roy and Chakravarty [31] in a survey of 557 new male entrants to medical schools in West Bengal found that 17.6% had a smoking experience and 3.2% were regular smokers. No female student out of a sample of 148 had ever smoked. Students from both urban and rural areas had the habit of smoking, but it was more common among the students originating from urban areas. Among the new entrants, those from Christian schools were more likely to smoke. Interestingly, there was no smoking habit among males from low income families. Another survey of 1600 medical students from the northern state of Uttar Pradesh found that the number of smokers increased with the increase in period of stay in the medical school [32]. Severity of smoking, as measured by the number of cigarettes smoked per day, also increased from first year to the final year in the school. Smoking habit of a family member seemed to influence the smoking habit of students, e.g., 68.2% of the family members (father, grandfather or brothers) were regular smokers, whereas in case of non-smokers this figure lowered to 18.36%. In another study on 672 medical students, Singh *et al*. found 26.4% to be smokers [33]. Only one third of them were smokers in the previous month. The number of smokers and the intensity of smoking increased with the advancement of their career in college. Smokers had a stronger family history of smoking.

**Table 2:** Studies on tobacco use among medical students

Author	Sample	Age of onset	Ever use	Current use	Current daily use	Type of tobacco use
Sinha & Gupta [5]	Male medical students (N = 400)	15–17	--	9.2% tobacco	43% tobacco; 12.5% **gutka**	Smoking and tobacco chewing
						
Venkatraman et al. [8]	Students of a medical college (N = 196)	Peak at Two years before and after joining the course	33.2%	--	--	Smoking
						
Roy & Chakravarty [31]	New admissions to medical colleges (N = 705, M 79.0%, F 20.0%) 90.1% in 17–20 age group	--	17.6% in males Nil in females	--	3.2% in males	Smoking
						
Sandell et al. [32]	Students of medical colleges (N = 1600)	--	26.5%; 26.2% in males 0.25% in females	--	--	Smoking
						
Singh et al. [33]	Students of a medical college (N = 672)Age group 17–24	--	Annual prevalence 26.4%	--	9.4%	Smoking

Venkatraman et al. studied time trends in smoking behavior among the alumni enrolled in MBBS course of a medical school in South India during the period 1955–1988 [8]. They identified three phases. In the first phase, there was a steady rise to peak between the last half of sixties and the early seventies. The prevalence seemed to plateau at a lower level in the next 10 years. However, a steep fall in smoking habit was noticed in the last five years of the study. The peak period of initiation seemed to be in two years before and after joining the course. There was a significant relationship between the presence of a smoker in the family and initiation of the habit. Quitting behavior was noticed at its peak 3 years after joining the course.

In another recent study on tobacco use among 400 male medical students from the state of Bihar in north India [5], 43% of the participants were regular tobacco users and a few (0.7%) areca-nut users. Occasional use of tobacco and areca-nut was found among 9.2% and 27.5% cases, respectively. Only less than 13% of the students were found to be aware of the harmful effects of different tobacco products. Nearly 50 (12.5%) students were regular users of manufactured smokeless tobacco products (MSTP) or *gutka*. Initiation of tobacco use peaked at class ten (32%) or classes 11 and 12 (32%), equal to the ages of 15–17. The factors found significant in leading to a higher tobacco consumption included lack of family supervision among hostels, peer influence, lack of awareness about the harmful effects of different tobacco products, no exposure to clinical cases of tobacco related disorders, and easy availability of these products.

It appears that from 1970 to 1998, there has been changes in patterns of tobacco use with a fall in cigarette smoking and an increase in MSTP consumption. This may be due to changes in social structure and behavior, increasing awareness about the hazardous effects of smoking and marketing effects of MSTP. Family influences remain an important factor among tobacco users.

### Tobacco use in street children

India has probably the largest population of street children in the world. The metropolitan cities like Delhi, Mumbai (Bombay), Kolkata (Calcutta) and Bangalore have each around 100,000 of street children. The majority of them maintain tenuous ties with their families, but many have no families at all, having an extremely stressful life.

Drugs of abuse give these kids excitement and relief from the all pervasive gloom of street life and serve to suppress hunger and helplessness. Tobacco-based products stimulating the CNS to improve alertness are used by these children to get relief from insomnia due to long working hours. The usual pattern of drug abuse by male and female kids of ages 5 to 10 is through chewing tobacco (**gutka **and **khaini**) mixed with other drugs as adulterants, followed by **beedis **and cigarettes. Older children of ages 15–16 use nicotine along with other hard drugs. There is scanty data on age at the first use of drugs, but anecdotal reports indicate that the age is very low and is getting further younger. The youngest age at the first use of a socially sanctioned drug has been reported to be five [34].

## Comparison with other Countries

The US studies show that 12.6% to 79% of the school students are current tobacco consumers, defined as having used a tobacco product on one or more days since the previous month [35-37]. Nearly, three fourth of them were found to be smokers. The figures show a rising trend from middle school to high school students [35]. Declining trends in smoking are evident among the school students over the last decade [37]. Tobacco addiction is equally common in both sexes [19].

According to the results of Global Youth Survey Project conducted on school children of age groups 13–15 in 12 countries (Barbados, China, Costa Rica, Fiji, Jordan, Poland, Russian Federation, South Africa, Sri Lanka, Ukraine, Venezuela and Zimbabwe), current cigarette smoking was found to vary from 10% to 33% and ever-smoking from 15% to 70% across different countries. In Russian Federation, Sri Lanka and Ukraine, smoking was found to be more common among boys than in girls, whereas it was more common among girls in China, Fiji, Jordan, and Venezuela [2].

Tobacco use and especially smoking is a male-dominated phenomenon among children and adolescents in India. This is unlike the West, where its distribution is equal among both genders. In some countries like China, Fiji, Jordan and Venezuela, smoking is rather more common among females. In India, the use of smokeless tobacco has become popular during the last few decades. A decline in tobacco use, as evident in the USA and some European countries, does not seem to be observed in near future in India. In contrast, the use of chewing tobacco seems to follow a rising trend, particularly among the street children and college students, which is a matter of serious concern.

## Psychosocial Factors Leading to Initiation of Tobacco Use

A number of factors influence the use of tobacco by children and teenagers. Some of these are the family history of tobacco use by elders, peer influence, experimentation, easy access to such products, personality factors, underlying emotional and psychological problems, accompanied risk-taking behaviors, and most importantly, the aggressive marketing strategies of the tobacco industry.

### The role of family

Family plays a very important role in initiation of tobacco use by a young child or adolescent. Tobacco use by parents or an elder sibling increases the likelihood that a child begins smoking [6,11]. As an example, many Indian fathers and grandfathers frequently ask the boys to fetch **beedis **or cigarettes from a nearby shop or kiosk. By this way, children are often introduced to such products at their very early life stages. A child growing in such a family watching his elder brother, father, uncles or grandfather using tobacco may perceive it as a family tradition that is to be followed. On the other hand, it is interesting to know that as an Indian tradition, younger individuals are not expected to smoke in the presence of elderly, because smoking by a younger person is taken as in contempt of the older people. Therefore, it is a paradox that the same elderly people, who passively show the way to smoke, are prohibitive of the same behavior by the younger generation in their own presence. However, this value system does not apply to the use of smokeless tobacco products.

### The role of peer influence

Although children may start smoking for psychosocial reasons like peer influences, curiosity, desire for experimentation or as a remedy for stress, the pharmacological motives take on place very early in their smoking career [39]. Consequently, by the time children smoke on a daily basis, they take up the same amount of nicotine from each cigarette as their adult counterparts do [40].

Peer pressure is an important determining factor for initiation of tobacco use among children and adolescents. There are several processes by which being associated with drug-using peers contributes to drug-abusing behavior. Here, modeling and social approval play an important role. When one is distressed due to any reason, an offered cigarette or **beedi **by a friend initiates the conforming process with a tobacco-using peer-group network [4,28].

### Easy availability of tobacco products

Tobacco products are socially sanctioned but are freely available in every nook and corner throughout the country. **Beedis **are a bit cheaper than the cigarettes and hence are preferred by the poor who cannot afford cigarettes. The newly introduced MSTP are also cheaper than cigarettes and do not carry the trouble of lighting, and therefore, are more convenient to use, which makes it popular among the users [5].

### Psychological/emotional factors

Poor school performance, truancy, low aspiration for future success, and school dropouts have been found to be associated with smoking at an early age [4]. Children and adolescents with anxiety and depression are likely to use tobacco and other drugs, as these have anxiety relieving and mood elevating properties [14]. Furthermore, such children may socially be anxious and feel isolated in a company of peer groups. Initiation of smoking helps them to identify with the group and hence reduces social anxiety [41]. Children with low self-esteem are likely to be vulnerable to drug use including the tobacco. As smoking behavior is associated with maturity and adulthood, tobacco use may serve to promote self-esteem [42].

### Promotion by tobacco companies

Advertisements of various tobacco products are very common in all forms of media including the print media, television, and the roadside hoardings and banners. Tobacco advertising and promotion effectively target the young people with images of smokers as trendy, sporty and successful [28,43]. Characters in the movies or television serials often demonstrate cigarette smoking as a routine of daily life. They sometimes even show cigarette lighting ways using different tricks. These scenes often attract the impressionable mind of the adolescent to use similar tricks or adopt similar behavior. For a child or an adolescent growing in a stressful home, television show and movies are a means of finding out what a normal life is about. He or she is likely to begin smoking after watching such visuals.

Some multinational tobacco companies are also involved in sponsoring of mega sport events like international cricket matches and some even have instituted bravery awards. These attract the vulnerable child or adolescent towards smoking with an idea that it may be a heroic or desirable activity to adopt because of the apparent positive associations. Advertisements and promotion of cigarettes have been reaching its peak in developing countries like India due to a sharp decline in sales in Western countries [2].

To summarize, multiple factors determine initiation of tobacco use. These factors are both promoting and prohibitive and have been constantly changing over the time depending on several economical and marketing forces. Traditionally, cigarette smoking had an association with the upper class of the society, reminiscent of the British colonial era. Though in the recent past it has percolated to all strata of the society, it is still more common among the upper and middle classes. Among the lower income groups, smokless tobacco like **khaini, mishri **or smoking **beedis **are common. In rural areas, smoking by **hooka **and tobacco chewing are common. Over the last 2–3 decades, particular chewable tobacco products are being highly promoted for sale and their use is being linked with the society's upper-class business-community in order to popularize them among the masses. Therefore, the socio-cultural influences are quite complex in these regards and in establishing changes in tobacco use among the public.

## Preventive Strategies

Considering the enormous adverse health consequences accompanying tobacco addiction, it is very important to develop preventive strategies to reduce tobacco consumption. Preventive strategies especially focused towards children and adolescents need to be initiated on emergent basis. This is more important for the developing countries like India, which have become the main targets of advertisement and promotional propaganda of various multinational tobacco companies [2,4].

Preventive approaches include spreading awareness about the actual hazards of tobacco in the community especially among the vulnerable children and adolescents, curbs on advertisement and promotional campaigns, early identification of the users and providing treatment [4,29,44,45].

### Early Education

The benefits of early educational programs have been well reported for school children [44]. Such programs should not only focus on the harms caused by cigarette smoking but also on those caused by other forms of tobacco use like smoking **hooka **and **beedis**, and by the smokeless forms like **gutka**. In fact, a majority of Indian people are not aware of the health consequences caused by smokeless tobacco products. Added to this, a wrong belief is being spread throughout the country that **beedis **are less harmful than cigarettes. Some indirect promotional efforts even spread an untruth about some beneficial effects of **beedis **such as being abdominal gas and constipation reliever! Thus, in a country like India, widespread community awareness program on the hazards of local and cheaper tobacco products like **beedis **and **gutka **are more essential than few school based programs.

A couple of programs are already in progress for the street children in India. A training module has been prepared for the non-governmental organizations (NGOs), for offering need-based intervention to the street children. The work was conducted under the auspices of WHO, UNICEF, and the Ministry of Health & Family Welfare of the Indian Government. Drug abuse and high-risk behavior have also been studied among the street children. UNICEF is conducting training courses for NGOs and street educators who are expected to find and help the street children abusing drugs [30,33].

### Curb on media advertisements and tobacco promotion

A very important step in primary prevention is the revamp of the existing tobacco policies of the government. The Government of India has recently taken some important legal measures, but there are still many problems in the enforcement of tobacco related law. Restrictions have been imposed on sales and on tobacco use in public places like railway stations, airports, hospitals and governmental offices. However, the more important aspect would be the strict observation and control of such restrictions. The sales of all tobacco products including the MSTP and their easy access strongly need to be banned for children and adolescents. An initiative in this regard has been taken by stopping tobacco sale in vicinity of schools. Countries like UK have imposed age restrictions in purchase of tobacco products. Stopping sales of tobacco to children is an important step towards reducing the number of tobacco users among the new generation [27].

### Overall community development

The limitations of the long-term success of the school-based interventions [45] have led researches to advocate approaches involving the creation of a wide social environment supportive of nonsmoking. This is extremely relevant to Indian societies, where economic disparity, unemployment, illiteracy and homelessness have been associated with all kinds of addictive behavior including the tobacco use by children.

### Legal Aspects

Tobacco industry has been facing strong opposition from the legal and public forums similar to those in the Western countries like USA. As the dangers of smoking began getting more and more identified, legal enforcements for prohibition of tobacco advertisements and declaration of nonsmoking zones have become a worldwide phenomenon, especially in the last 2–3 decades. India has recently passed the Tobacco Products Bill 2001 [35,46], which prohibits advertisement of all tobacco products, smoking at public places and selling tobacco to persons under 18 years of age. It also directs the manufacturers of tobacco products to indicate the nicotine and tar contents and warnings of adverse health effects of tobacco products on packages in both English and regional languages. In addition, the bill places a total ban on sponsorship of any sport or cultural events by tobacco companies. During the last 5 years, the State Governments have also passed relevant orders for smoking ban in public places. One such order prohibits the sale of tobacco products within 100 m of schools [29].

The effectiveness of many of these steps is now well documented. Long term studies have shown that a ban on advertising tobacco products is effective in reducing their consumption [47]. It is also important that an addictive product associated with serious diseases and death, must not be allowed to be presented to public through sexy, glamorous and macho images [35].

Enforcements of laws have often been a problem in India. For example, although nonsmoking zones exist at important public places, especially in the large cities, there is an utter disregard for them. Nobody minds smoking by others, particularly in villages or small cities. There is a need to initiate massive public awareness campaigns educating people about serious health consequences of smoking, passive smoking, and smokeless tobacco use. Steps towards this direction have been taken, but it may take some time to obtain the results.

## Conclusion

Humans have used tobacco in many forms for several centuries. Its use often starts early in life. In recent years, there has been a rising trend in tobacco use, more in smokeless forms in India. There are no nationwide data available in India on the exact extent of the tobacco use among adolescents, although a number of surveys have been reported from different parts of the country. These show a general tendency towards an increase in tobacco use by the youth for the past few decades, with an emphasis on the use of smokeless tobacco. This is a matter of great public health concern. Psychosocial factors have an important role to play in initiation of this habit. It has been observed that a large number of adolescents pick up this habit from their family members or the peers. Advertisements of tobacco products and promotional campaigns by the manufacturers also play an important role in initiation of the habit by adolescents. This has attracted the attention of health professionals, media and law enforcement agencies. The local governments are also taking steps in putting curbs over the sales of tobacco products to children, and in regulating tobacco advertisements.

There is a need to collect nationwide data on the use of different forms of tobacco by children and adolescents, and the factors leading to initiation of such harmful habits. There is an urgent need to take effective steps, especially on launching community awareness programs for the school children and public to educate them about the consequences of tobacco use, and on assessing their effectiveness in curbing the problem. It is also necessary to keep abreast of the policies and conventions of the international agencies like WHO, UNDCP and other similar agencies on tobacco use, in order to utilize their expertise for curbing this problem.

## Note

* English medium schools are the ones where English is the primary language of instruction for all subjects except for the modern Indian languages, whereas in Indian language schools, one of the Indian languages is the language of instruction for different subjects. Municipal schools are the ones run by the State, whereas the private schools are run by private organizations and not financed by the State.

## Competing interests

The authors declare that they have no competing interests.
